# Inclusive, supportive and dignified maternity care (SDMC)—Development and feasibility assessment of an intervention package for public health systems: A study protocol

**DOI:** 10.1371/journal.pone.0263635

**Published:** 2022-02-09

**Authors:** Bilal Iqbal Avan, Waqas Hameed, Bushra Khan, Muhammad Asim, Sarah Saleem, Sameen Siddiqi

**Affiliations:** 1 Department of Clinical Research, London School of Hygiene and Tropical Medicine, London, United Kingdom; 2 Department of Community Health Sciences, Aga Khan University, Karachi, Pakistan; 3 Department of Psychology, University of Karachi, Karachi, Pakistan; PLOS: Public Library of Science, UNITED KINGDOM

## Abstract

**Introduction:**

Mistreatment, discrimination, and poor psycho-social support during childbirth at health facilities are common in lower- and middle-income countries. Despite a policy directive from the World Health Organisation (WHO), no operational model exists that effectively demonstrates incorporation of these guidelines in routine facility-based maternity services. This early-phase implementation research aims to develop, implement, and test the feasibility of a service-delivery strategy to promote the culture of supportive and dignified maternity care (SDMC) at public health facilities.

**Methods:**

Guided by human-centred design approach, the implementation of this study will be divided into two phases: development of intervention, and implementing and testing feasibility. The service-delivery intervention will be co-created along with relevant stakeholders and informed by contextual evidence that is generated through formative research. It will include capacity-building of maternity teams, and the improvement of governance and accountability mechanisms within public health facilities. The technical content will be primarily based on WHO’s intrapartum care guidelines and mental health Gap Action Programme (mhGAP) materials. A mixed-method, pre-post design will be used for feasibility assessment. The intervention will be implemented at six secondary-level healthcare facilities in two districts of southern Sindh, Pakistan. Data from multiple sources will be collected before, during and after the implementation of the intervention. We will assess the coverage of the intervention, challenges faced, and changes in maternity teams’ understanding and attitude towards SDMC. Additionally, women’s maternity experiences and psycho-social well-being—will inform the success of the intervention.

**Expected outcomes:**

Evidence from this implementation research will enhance understanding of health systems challenges and opportunities around SDMC. A key output from this research will be the SDMC service-delivery package, comprising a comprehensive training package (on inclusive, supportive and dignified maternity care) and a field tested strategy to ensure implementation of recommended practices in routine, facility-based maternity care. Adaptation, Implementation and evaluation of SDMC package in diverse setting will be way forward. The study has been registered with clinicaltrials.gov (Registration number: NCT05146518).

## Introduction

The experience of childbirth can change a woman’s life and leave lasting memories [1, 2]. Up to a third of women appraise their experience of giving birth as traumatic [3]. These psychological distresses during labour leaves them vulnerable to the care processes in health facilities such as unfamiliar personnel, medicalised procedures and other conditions [4]. Regardless of it, women around the globe, particularly in low- and middle-income countries (LMICs) are mistreated and rarely offered psychosocial support during childbirth at health facilities [5]. Such poor encounters may have adverse effects on the quality and outcome of birthing experiences, which could lead to psychological disorders [6–9], and may deter women from seeking health care in the future [10]. Additionally, range of other socio-economic (e.g. poverty) and health-related (e.g. anxiety, depression or disability) vulnerabilities predispose women to discriminatory care which may further aggravate the situation, leading to more intense sufferings [11].

Respectful Maternity Care (RMC) not only reduces the need for superfluous medical intervention but also strengthens psychosocial processes, thus improves birth outcomes and maternal well-being [4]. In the year 2014, the World Health Organisation (WHO) issued a policy statement that “every woman has the right to the highest attainable standard of health, which includes the right to dignified, respectful health care” [12]. Subsequently, they released a revised framework for quality of maternal and newborn healthcare that put special emphasis on women’s experience of care (dignity, effective community and emotional support) [13], followed by another framework to aid understanding of different forms mistreatments encountered by women during childbirth [5]. More recently, WHO released a comprehensive set of evidence-based policy recommendations to promote women’s experiences of intrapartum care [14]. These policy directives from the WHO indicate the need for integrated health services that should not only respect women’s dignity but also fulfil their emotional or psychosocial needs during maternity care [12]. This proposition presents with an opportunity to link WHO’s Mental Health Gap Action Programme (mhGAP) materials in addressing psychosocial needs of pregnant women by bringing a positive change in the maternity care settings, as per newly set WHO standards for intrapartum care [14].

However, despite the release of these guidelines, no operational model exists that effectively demonstrates incorporation of these principles in routine facility-based maternity services [15]. Strategies applied thus far have not been adequately embedded in health systems. While these approaches might appear effective, information about them is relatively sparse, and their overall evidence is rated with low certainity for risk of bias for all outcomes [15]. Moreover, these interventions have suffered from numerous challenges around acceptability and feasibility, particularly in low-resource settings where the health system was plagued with issues of governance and accountability, and mistreament of women was considered socially acceptable [10, 16]. It is therefore recommended that, in order to achieve greater feasibility, acceptability and impact, the design and development of such interventions should use a participatory approach, while taking into account the socio-cultural context and the health system’s environment and constraints [16].

Keeping in view of the complexity of the issue, translation of WHO’s policies and guidelines into practice necessitates the use of implementation research to inform design and implementation of contextually-appropriate interventions, and embedding them within the healthcare system in a sustainable manner. Following Medical Research Council framework, we propose early-phase implementation research to develop, implement and test feasibility of a service delivery intervention package before running a full-scale trial [17].

### Aim and objectives

**Fig 1:**
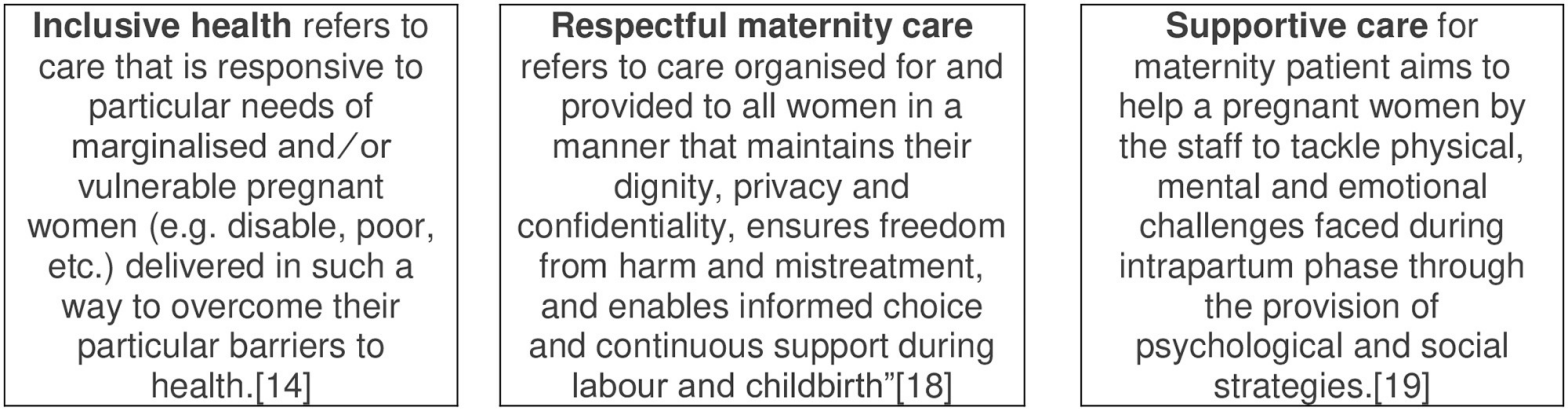
Operational definitions of key concepts [14, 18] a[19].

The overall aim of this research is to support inclusive, supportive and dignified maternity care within health systems, especially from the perspective of a) capacity of service providers, and b) accountability and governance (see Fig 1 for definition of key concepts). The specific objectives are:

a) Design a contextually-informed (or evidence-based) service-delivery model through a participatory, consensus-driven process for inclusive, supportive and dignified maternity care; and
b) Implement and test the feasibility of the intervention model for its content and operationalisation in public health facilities

## Methods

### Country context

This early-phase study will be conducted in Pakistan which has one of highest rates of maternal mortality [20], and is ranked the riskiest country for newborns [21]. The country also has the highest prevalence (range 29–66%) of anxiety and depression among women in Asia [22]. Healthcare in Pakistan is delivered through a three-tiered health system: primary, secondary and tertiary. The proposed research focuses on secondary-level healthcare where inpatient childbirth facilities are formally introduced through maternity ward and labour room services. Specialist consultation and hospital admissions fall into this category. It includes two types of health facilities: district (District health quarter Hospitals–DHQ) and sub-district level (Tehsil Head Quarter—THQs). Recent evidence from secondary-level health facilities revealed high reports (97%) of disrespectful and abusive intrapartum care in health facilities, including a violation of women’s right to information (70%); lack of consensual (78%) and confidential care (69%); lack of supportive care; loss of women’s autonomy; and verbal and physical abuse [23]. The proportion of total births that takes place in public health facilities is startlingly low at (15%) [24]. Numerous factors in the running of health systems contribute to this compromised care, among them: lack of awareness and professional capacity of service providers around respectful and supportive care; a non-conducive work environment; and poor governance and accountability [25].

### Intervention

It will be a theory-driven, inclusive service-delivery package which will be developed using the principles of the human-centred design approach. The model will include capacity-building of maternity teams, and the improvement of governance and accountability mechanisms within public health facilities to ensure that all women are treated with compassion and dignity, while also catering for their diverse needs, including disabilities and common mental health conditions. The integration of psychosocial support in routine maternity care will be a unique feature of the intervention package, a principal aim of which is to address the psychological needs of birthing women and their companions. The technical content of the SDMC training handbook will be based on two integrated modules: WHO’s intrapartum care guidelines [14] and Mental Health Gap Action Programme (mhGAP) training [26] and implementation guidelines [27]. As part of the SDMC package, maternity staff will have job-aids for referencing of essential SDMC behaviours, steps of providing psychosocial support, and reassuring maternity patients about their rights and responsibilities.

Operationally, the core components of intervention would include: capacity-building of a typical maternity team comprising clinical staff (gynaecologists, doctors, nurses) and support staff (ward boys/porters and maids); collaborative care, a team-driven approach, led by gynaecologists (ward in-charge), attempts to integrate care and behaviour practices of staff according to the principles of measurement-guided care-plans, quality improvement and accountability, with a focus on meeting SDMC needs; staff and patient feedback: a complaints register will be maintained by designated staff; and brief exit interviews will elicit, and record, the degree to which care has been supportive and respectful; Accountability and governance (performance review): reports of non-respectful care will be discussed, and remedial actions decided, in periodic meetings.

### Theory of change

The intervention development will be guided by the COM-B (’capability’, ’opportunity’, ’motivation’ and ’behaviour’) framework. Conceptually, the intervention will aim to improve *capability* of care providers to provide supportive and dignified care by providing relevant knowledge and skills, *opportunity* to enact such behaviours by altering changes in the systems and inculcating the use of collaborative care approach, and *motivation* by re-enforcing the benefits of inclusive care on individual, health systems, and community [28].

**Fig 2:**
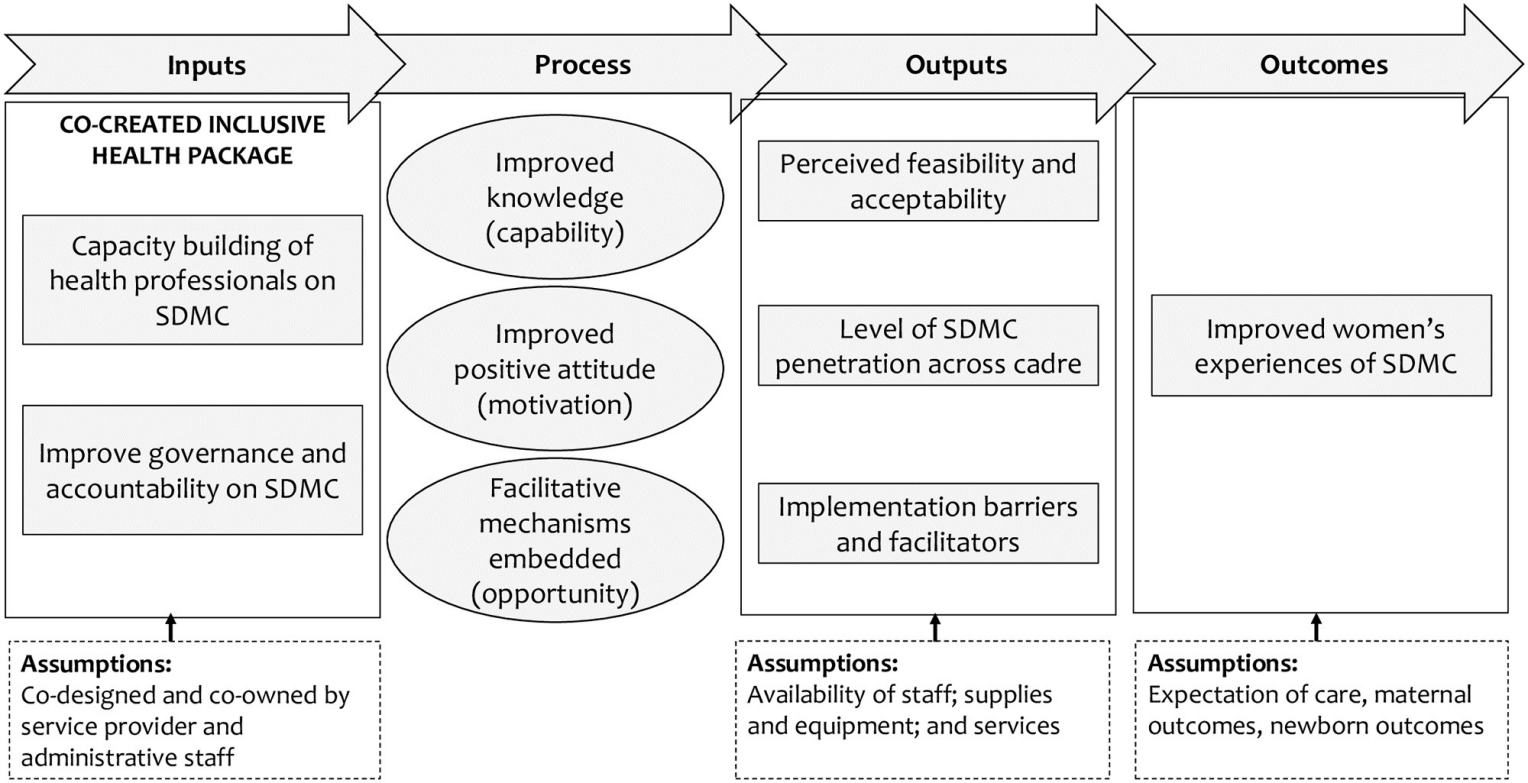
Logic model of the research.

It is hypothesised that building capacity for collborative care, improving maternal orientation, and embedding review and accountability mechanisms in health facilities will enable service providers and staff to provide supportive and dignified maternity care. This will lead to improvement in health systems’ overall responsiveness towards psychosocial needs of women and ensuring women’s dignity is respected (see Fig 2 for logic model).

### Study phases

#### Implementation phases

The implementation of this study will be divided into two phases: (1) Development of intervention: grounded in formative research around supportive and dignified care, participatory approach will be used to co-create service-delivery intervention package; and (2) Implementing and testing feasibility: a two-level feasibility assessment will be carried out–health systems and staff assessment, and women’s experiences around SDMC.

**Fig 3:**
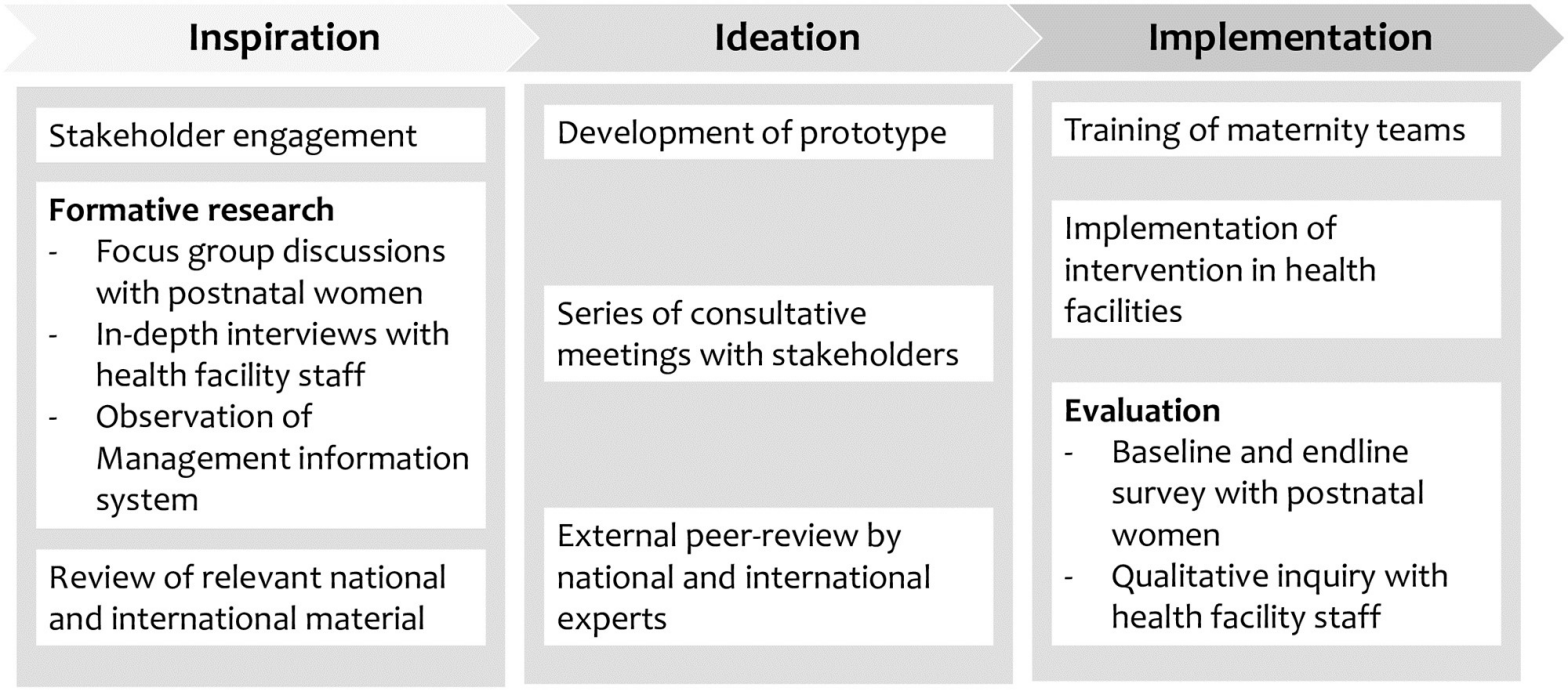
Stages of SDMC intervention development, implementation and feasibility testing.

We will use the framework of human-centred design (HCD) to guide implementation of this study (see Fig 3). Human-centred design is an emerging concept and being used increasingly in global health [29]. The approach is classified in three phases: inspiration, ideation, and implementation and testing.

### Objective 1—Design a contextually-informed (or evidence-based) service-delivery model through a participatory, consensus-driven process for inclusive, supportive and dignified maternity care

#### Phase 1: Inspiration

The first step is empathise–whereby, through a formative research we will understand prevalent behaviours, challenges and opportunities around SDMC. It will be supplemented by review of national and international guidelines, and best practices around the topic in focus. We will begin with the participatory approach to engage with key stakeholders, including practitioners, programme managers, policy makers, and community representatives to ensure contribution and ownership of the project.

#### Formative research

*Focus Group Discussions (FGDs)*. A total of five to eight FGDs will be conducted with postpartum women who have delivered in public health facility during the last three months. These women will be identified from a list frame to be obtained from the selected health facility. The FGDs will broadly explore: (a) attitudes towards facility-based births; (b) motivations to give birth at the facility; (c) expectations regarding maternity care; and (d) perceived quality of care with according to the Hulton framework for quality of care in maternity services [30]. The framework focuses on both ‘provision of care’ and ‘experience of care’ (human and physical resources, cognitive support, respect and dignity, equity, emotional support). A trained sociologist/anthropologist will conduct FGDs who will be accompanied by note-taker. Informed consent will be taken from participants of FGDs and all discussions will be audio-recorded. The data generated from FGD will inform development of SDMC intervention package and contextualised structured tools to assess experiences of SDMC at healthcare facilities.

*Health facility assessment*. The assessment of health facility will comprise of: a) interviews with health system staff (clinical and non-clinical service providers and administrators) and b) review of management information system.

- ***Indepth interviews (IDIs) with staff*:** IDIs will be conducted with clinical and non-clinical maternity staff and administrators to broadly understand challenges and opportunities around SDMC. More specificially, our themes would include: routine operations of maternity section, governance and accountability mechanisms around SDMC; knowledge, attitude and motivation towards SDMC; and experiences of patient interactions. Additionally, we will explore their perceptions about working conditions, job responsibilities, empathy with and prejudice against patients, and relationship with co-workers and supervisors. Preliminary information about health management information system will also be collected during these interviews and will be systematically assessed in observation visits. The COM-B behavioural framework will guide the the development of interview guide and data synthesis to understand individual and systemic factors around motivation, capabilities and opportunities for supportive and dignified maternity care.

*Observations*.

- ***Review of management information system (MIS)*:** We will review the kind of information documented on inpatient records and assess mechanisms to use this data to make informed decisions. Understanding MIS will enable the investigators to create or strenthen mechanism for record keeping on incidents of any mistreatment as a measure of accountability.

#### Review of relevant national and international material

We will conduct a thorough review of existing national and international material that is pertinent to the supportive and dignified maternity care. This would broadly include: existing national service delivery guidelines for maternity care during labour and childbirth; WHO recommendations for intrapartum care; mhGAP material for neurological disorders and substance abuse; existing training material on respectful maternity care.

#### Phase 2: Ideation

In second phase, data synthesis will generate numerous issues and their possible solutions around supportive and dignified maternity care. These solutions/ideas will then be discussed with stakeholders to select the ones that are most relevant and applicable in the given context, around which the prototype will be formally developed. The prototype will undergo provisional testing through a collective review with stakeholders–who will reflect on its relevance and operational feasibility in their routine practice. The prototype will then be revised based on feedback and learnings accordingly. If the agreed solutions do not seem to be viable, the new sets of ideas will be explored altogether from data and in brainstorming sessions with stakeholders. This will be iterative process will run till the group reaches to a final consensus.

### Objective 2: Implement and test the feasibility of the intervention model for its content and operationalisation in public health facilities

#### Phase 3: Implementation and testing

The third phase of HCD fall within the scope of second study objective. In phase three, the finalisation of prototype will be followed by pilot testing in real-life settings over a period of three months. It is important to note that this implementation is will be considered pilot as the field learnings will be fed back into design of intervention package.

*Evaluation design*. A mixed-method, pre-post design will be used to test feasibility of the intervention prototype.

*Study settings*. The study will be implemented at six public health facilities–with three facilities in each Thatta and Sujawal districts of southern Sindh province, Pakistan. These contagious districts are located approximately 100 kilometres away from megacity, Karachi. Each district has a population of approximately 0.8 million inhabitants, with 82% of them residing in rural areas [31] and just over half (52.3%) of the population belong to the poorest wealth quintile relative to the Sindh province (South of Pakistan) [32]. Only 15.8% of the total births take place in public sector health facility [32].

*Study population*. The primary target-audiences will be women in childbirth and service providers (clinical and non-clinical staff) of the labour room / maternity wards.

#### Health system and staff assessments

*Quantitative methods*. Baseline assessment will follow the capacity-building of clinical and support staff of the health facility in training modules. The maternity team will receive comprehensive 3-day training on SDMC that is tailored to their contextual settings. Senior staff members will become master trainers, responsible for providing supportive supervision to ensure adherence to set standards. Feedback from staff will be sought during the training about the relevance of content and its implementation feasibility.

The knowledge and attitude of training participants (clinical and non-clinical staff and administrators) regarding SDMC will be assessed through pre-and post-training tests. The tools will be developed in accordance with the intervention package, after it is finalised.

*Qualitative methods*. During the intervention period quality audits (or supportive supervision visits) will be routinely conducted on all selected health facilities to assess adoption of, and adherence to, quality standards through observation of women-provider interactions, staff coordination, and facility assessments. The maternity staff will be provided with customised feedback based on the observation findings.

Post-intervention, in-depth interviews will be conducted with practioners and programme managers to explore intervention fidelity, dose and coverage, penetration of SDMC practices across cadre, and individual, interpersonal, and system-level facilitators and barriers that were encountered during delivery of the intervention in routine practice. We will also gather their suggestions for improvement in the SDMC intervention package.

#### Women’s experiences

*Quantitative methods*. Pre-post intervention assessments will gather information on women’s experiences of SDMC at health facilities, and suffering of postpartum depression within 42 days of childbirth. Unlike health system assessment, two independent group of women will be enrolled in baseline and endline. Recently published structured questionnaire on mistreatment during childbirth will be contextually adapted and used for these pre-post assessments [33]. Postpartum depression and anxiety will be assesed using Patient Health Questionnaire– 9 and Generalised Anxiety Disorder– 7, respectively.

*Sample size calculation*. In order to get a sense of sample size requirement for a meaningful change study outcomes, the sample size was estimated with varied effect sizes. Mean score mistreatment was considered as key outcomes which is taken from a background study in similar setting that yielded a mean score of 25 (score range 0 to 40). A total of 308 postpartum women will be required to detect a statistical difference of 2.5-units decrease (or 10% reduction) in the mean score of mistreatment (from 25 [34] to 22.5) with standard deviation of 6 [34], 90% power, 5% level of significance, 2 design effect, and 10% lost-to-follow-up (LTFU) and/or non-response. We decided to proceed with a sample of 308 as it gives sufficient precision to perform range of additional analysis such as estimation of prevalence of different types of mistreatment and the effect of intervention on each of these types of mistreatment, while will not negatively affecting study timelines.

*Sampling strategy*. A convenience sampling technique will be used for the selection of study participants. All women giving births at the selected hospitals during the data collection period will be invited to participate in the survey, until the desired sample is achieved. The volume of women attending each health facility for childbirth may vary; hence based on historical trends the sample size will be proportionally distributed across health facilities according to the caseload. During the consent process, participants will be informed and asked whether they would be interested in participating in a follow-up interview in their homes at six weeks of postpartum. Women who showed interest in home-based interview and gave consent will be recruited in the survey. Participants who lived outside of the study district or even in hard-to-reach rural areas of the district will be excluded from this sample due to logistical constraints.

*Data collection and management*. Standardised and contextually-adapted tools will be used in this study. All data collection tools will be translated in the relevant local language and pre-tested in similar settings prior to being used. Data collectors with prior experience will be hired and trained for baseline and endline assessments, and routine audits. In-depth interviews (IDIs) and focus group discussions (FGDs) will be conducted by trained sociologist and/or anthropologist. All data collectors will receive a comprehensive and standardised training on both theoretical and practical aspect of study instruments. In addition, data collectors will be trained on rapport building, research ethics, and interviewing techniques. Research Officer will conduct: shadow interviews (observation of data collection) and independent re-interview/assessment with a sub-sample to ensure data quality.

Qualitative interviews will be audio-recorded for later transcription and translation. All quantitative data will be collected electronically on tablets. The software application, developed in Epicollect5, will have built-in validation checks to minimize data collection and entry errors. All software applications will be tested prior to their use in the study. Unique IDs will be given to each study participant which will be consistent (where applicable) in across assessments for synchronisation. Data Management person, under the supervision of study Co-Investigator will be responsible to manage all data that include: timely transfer to central database, data quality through routine assessments; data confidentiality, security, back-up, and sharing while maintaining anonymity. Study Investigators will coordinate closely with research officer and data collectors to ensure timely remedial measures.

*Data analysis*. Descriptive statistics will be used to describe the characteristics of study participants such as age, level of education, socio-economic status etc. In order observed change in women’s experiences of SDMC, advanced statistical techniques will be used to estimate changes in outcome indicators (SDMC experience) by means of multivariable linear (continuous) and logistic (binary) regression models, accounting for intra-class correlation within health facilities. We will create a composite score for women’s experiences of SDMC which will treated as outcome for pre-post comparison using linear regression. Depending on distribution of the variable, we will consider dichotomising it to see pre-post intervention changes in prevalence of SDMC experiences using logistic regression. Paired t-test (for continuous) and McNemars test (for binary) will be used to observe change in knowledge and attitudes of health facility staff before and after training. Stata version 16.1 (StataCorp LP, Texas, United States) will be used for all analyses, and p-values of 0.05 will be considered statistically significant.

For the qualitative enquires of formative research, we will use bottle-neck analysis approach to identify prevalent issues that impedes provision of SDMC in health facilities, and also determine the opportunties that can be leverage to promote SDMC. These findings will inform the development of intervention package. The qualitative data to be collected post-intervention, will be analysed to understand feasibility and acceptability of SDMC intervention, and related barriers and facilitators of implementation. We will also analyse suggestions of study participants to make improvements in the intervention package.

The researchers will thoroughly review all of the transcripts several times to identify predetermined and emerging themes from the data as per the objectives. Audio-recording of the IDIs will be transcribed verbatim and translated from Urdu/local language into English. These transcripts will be used for detailed analysis. The data will be coded and thematically analysed using QSR NVivo 11 software for Windows.

*Covariates*. The adjustment for potential confounders will be primarily required to see changes in women’s experiences of SDMC. The analysis will be adjusted for following covariates: women’s age, ethnicity, level of education, employment status, level of empowerment, socio-economic status, functional disability, and anxiety or depression. These characteristics have found to be associated with disrespectful and abusive care at health facilities [5].

The progress and success of this early-phase research will be monitored and evaluated based on key indicators presented in Table 1. The immediate outputs will be development of a training manual for health facility staff and their training which will lead to improved knowledge and positive attitude towards regarding SDMC. Simultaneously, changes in the routine system will take place to create opportunities and encourage them to practice new behaviours. Post-intervention, perceived feasibility, penetration and challenges will be assessed that inform success of the intervention.

**Table 1:** List of input, process, intermediary and outcome indicators.

Level of indicator	Indicators	Instrument/ means of verification	Time	Objectives
Inputs	SDMC training handbook finalised based on the feedback of feasibility testing	Training manual	Inception phase	Objective 1
	Structural changes incorporated within health system	Systemic changes	Inception phase	Objective 1
	Number of participants trained on SDMC	Training reports	Inception phase	Objective 2
Intermediate outcomes	Mean score of service providers’ knowledge of SDMC	Knowledge questionnaire	Pre-post training test	Objective 2
	Mean score of service providers’ attitude towards SDMC	Attitude questionnaire	Pre-post training test	Objective 2
Implementation/ process outcomes	Penetration of SDMC across cadre (qualitative)	in-depth interview	Endline	Objective 2
	Feasibility and acceptability of SDMC package (qualitative)	in-depth interview	Endline	Objective 2
	Barriers and facilitators to implementation of SDMC (qualitative)	in-depth interview	Endline	Objective 2
Service outcomes	Mean score of women’s experiences of inclusive, supportive and dignified maternity care	Structured SDMC questionnaire	Baseline and endline	Objective 2

#### Key indicators

*Ethical considerations*. The research will be implemented in adherence to all ethical principles; and study documents will be reviewed and approved by relevant ethics review committees. The project team is committed to ensure that this research proposal and the actual implementation of the proposed research project adhere to core standards in research ethics. Informed consent will be obtained separately from all research participants. All study participants, irrespective of whether information is being elicited using qualitative or quantitative methods, or observation, will be informed about the purpose of study, potential risks and benefits it may cause, their right to refuse to answer any question and right to abstain from participating in the study, or to withdraw from it at any time with reprisal. Informed written consent will be taken from all study participants. For study participants who are illiterate, participant information sheet will be read out to them in presence of a witness–who will be a trustworthy person, not belonging to the study team. Our target audiences will be health facility staff and postnatal women which do not fall into the category of minor. However, it is possible that a woman may be under the age of 18 years. In case, she will be considered as emancipated minor and hence she can give informed consent for herself. Thumb impression will be taken from illiterate participants along with a signature from their witness. The informed consent process will be conducted in a manner that is appropriate for the local language, literacy level, and comprehension level of the target population. To ensure confidentiality and anonymity, all interviews will be conducted in privacy; data will be kept in password protected server; and participant’s contact details will be separate from data.

The study protocol, the informed consent forms, and other appropriate documents will be submitted and approved as required by Ethics review committees of the Aga Khan University and Research Ethics Committee to London School of Hygiene and Tropical Medicine. All research staff will be trained in research ethics. The Principal Investigator (PI) at the research site will be responsible for ensuring that all requirements of the local ethics committee are met. Before implementing any changes to the protocol, informed consent, advertising, or participant written materials, the site PI will have the changes approved by the local ethics committees, except where necessary to eliminate immediate hazards to human participants.

*Status and study timelines*. This study will span over twenty four months. The first three months will involve planning, development of study protocol and draft of study questionnaires, obtaining ethical clearance, and hiring of project staff. The next four months will be used for formative research to inform the design and development of SDMC intervention. It will follow development of SDMC intervention and its implementation strategy through a consultative process with stakeholders over six months period. The finalization of intervention package will be followed by baseline assessment and training of service providers over two months. Intervention will run over a period of six months followed by three months of endline assessment, data analysis, writing, and dissemination.

## Discussion

Women’s experiences of care within health system is an integral component of maternal and newborn quality of care framework [13]. Supportive and dignified maternity care places woman at the centre of decision making and providing her information regarding the process, and focuses on the interpersonal aspect of care. Our research aims to provide evidence of the successful operationalisation of supportive and dignified maternity care package by public health systems in low- and middle-income countries. This research becomes crucially important in the absence of any effective service-delivery models that support the implementation of WHO-recommended guidelines around supportive and dignified care in facility-based maternity services.

Evidence from this early-stage research will enhance understanding of health systems challenges and opportunities around SDMC. A key output from this research will be the SDMC service-delivery package, comprising a comprehensive training package (on respectful and supportive maternity care) and a tested strategy to ensure implementation of recommended practices around supportive and dignified care in routine, facility-based maternity care. There will also be sustained support and facilitation for practitioners and programmers throughout the implementation of SDMC standard guidelines in routine, facility-based maternity care. Furthermore, to ensure wide-spread access to the knowledge generated from this research, policy/research briefs will be written, flyers developed, and dissemination events organised for diverse audiences, including women, practitioners, managers, and policy-makers.

It is pertinent to note that this early-phase research so it will not provide robust evidence on the effectiveness of the intervention such as change in women’s experiences. The primary reason being that it has a greater focus on intervention development and testing its operational feasibility, while anticipating a change in women’s experiences is not the primary objective. Secondly, the intervention period is very limited which perhaps is not sufficient to smoothly embed and run systemic changes, and changing individuals’ behaviours.

Our research findings will directly benefit the Department of Health, followed by policy-makers and health-system programmers at the national level. Health-managers and policy-makers will develop an understanding of health-system issues that impede the provision of respectful and dignified care. Our findings will serve as the basis for formulating evidence-informed standards and policies that ensure the provision of humanised, respectful, and rights-based care, complemented by psycho-social support. Moreover, the engagement of international experts for peer-review of intervention material will not only improve the quality of the intervention and aligning it with global agenda, but it will also create avenue for further collaborations by testing its effectiveness in diverse settings in multiple countries.

The secondary output of this research would include: contextually-adapted measurement tools for SDMC and publication of methodological papers that would guide development of health-system interventions in collaboration with direct beneficiaries, in LMIC settings. Based on sound feasibility—especially its potential to improve maternity-ward processes and practices, and mothers’ intrapartum-care experiences—our aim would be to evaluate the effectiveness of the SDMC package through cluster randomized control design and scalability in various contexts.
